# An unusual new centipede subgenus *Lithobius* (*Sinuispineus*), with two new species from China (Lithobiomorpha, Lithobiidae)

**DOI:** 10.3897/zookeys.980.47295

**Published:** 2020-10-28

**Authors:** Xiaodong Chang, Sujian Pei, Chunying Zhu, Huiqin Ma

**Affiliations:** 1 Institute of Myriapodology, School of Life Sciences, Hengshui University, Hengshui, Hebei 053000, China Hengshui University Hengshui China; 2 Hebei Key Laboratory of Wetland Ecology and Conservation, Hengshui, Hebei 053000, China Hebei Key Laboratory of Wetland Ecology and Conservation Hengshui China

**Keywords:** Chilopoda, Lithobius (Sinuispineus) minuticornis sp. nov., Lithobius (Sinuispineus) sinuispineus sp. nov., Myriapoda

## Abstract

The present study describes a new Lithobiomorpha subgenus, Lithobius (Sinuispineus)**subgen. nov.**, and two new species, L. (Sinuispineus) sinuispineus**sp. nov.** and L. (Sinuispineus) minuticornis**sp. nov.** from China. The representatives of the new subgenus are characterized by a considerable sexual dimorphism of the ultimate leg pair 15, having the femur and tibia unusually enlarged in males, and the dorsal side of the femur with curved posterior spurs. These features distinguish Lithobius (Sinuispineus)**subgen. nov.** from all other subgenera of *Lithobius*. The diagnosis and the main morphological characters of the new subgenus and of the two new species are given for both male and female specimens.

## Introduction

Located in the east of the Asian continent, on the western shore of the Pacific Ocean, the People’s Republic of China has a land area of approximately 9.6 million square kilometres, and is the third largest country in the world (Zhang 2011).

Currently, the World Catalogue of Centipedes (Chilopoda) (Bonato et al. 2016) includes more than 1,200 valid species of Lithobiomorpha in nearly 130 extant genera and subgenera in only two families. However, the myriapod fauna of China is still poorly known, and very little attention has been paid to the study of Lithobiomorpha, which has only 85 species and subspecies (Pei et al. 2019; Qiao et al. 2019a, 2019b) currently known from the country. Knowledge of Chinese Lithobiomorpha is very fragmentary, and many species records and descriptions are widely scattered in the faunistic and taxonomic literature (Ma et al. 2014). A new subgenus and two new species have recently been discovered when examining material of Lithobiomorpha from Fujian and Henan provinces. The description of these new taxa is given below.

## Materials and methods

Specimens were collected under leaf litter or stones and preserved in 75% ethanol. Illustrations and measurements were produced using a ZEISS SteREO Discovery.V20 microscope equipped with an Abbe drawing tube and an ocular micrometre and Axiocam 512 colour 12-megapixel microscope camera. The colour description is based on specimens fixed in 75% ethanol. The body length was measured from the anterior margin of the cephalic plate to the posterior end of the postpedal tergite. Type specimens and other material are deposited in the School of Life Sciences, Hengshui University, Hengshui, China (HUSLS). The terminology of the external anatomy follows Bonato et al. (2010). Measurements are shown in millimetres (mm). The following abbreviations are used in the text and the tables:

**a** = anterior;

**C** = coxa;

**F** = femur;

**m** = median;

**P** = prefemur;

**p** = posterior;

**S, SS** = sternite, sternites;

**T, TT** = tergite, tergites;

**Ti** = tibia;

**Tr** = trochanter.

## Taxonomy

**Class Chilopoda Latreille, 1817**

**Order Lithobiomorpha Pocock, 1895**

**Family Lithobiidae Newport, 1844**

### Genus *Lithobius* Leach, 1814

#### Subgenus Sinuispineus subgen. nov.

Taxon classificationAnimaliaLithobiomorphaLithobiidae

6DBFD633-8975-5406-A923-18D69CC4A4F6

http://zoobank.org/35BCDB14-C94F-4E06-BAEC-1488605FCB4E

##### Type species.

Lithobius (Sinuispineus) sinuispineus sp. nov.

##### Diagnosis.

Sinuispineus subgen. nov. differs from the other subgenera of *Lithobius* in having curving posterior spurs on dorsal side of the femur of male leg 15; the prefemur and femur and tibia of male leg 15 markedly incrassate; prefemur and femur and tibia of male legs 14 also thicker than legs 1–13. Antennae 20–25 articles, 9–13 ocelli in three irregular rows, posterior two ocelli comparatively large; Tömösváry’s organ larger than the adjacent ocelli; prosternal teeth commonly 2+2, rarely 3+3; posterior angles of all tergites without triangular projections; coxal pore formula 3–6 in one row; tarsal articulation ill-defined on legs 1–13, well-defined on legs 14 and 15; female gonopods with 2+2 moderately small coniform spurs; male gonopods short and small.

##### Etymology.

To emphasize the obviously curved posterior spurs on the dorsal side of the femur of the male leg 15.

##### Distribution.

Fujian and Henan provinces, China.

##### Remarks.

Lithobius (Sinuispineus) is identified as a member of Lithobiidae based on the following: forcipular pleurites not meeting ventrally, male gonopods not visible, 9–13 ocelli, antennomeres 20 or thereabouts, posterior angles of all tergites rounded, spiracle lacking on the first leg-bearing segment, spurs lacking on tibia, and at least some legs with regularly disposed distal spurs on various articles. Lithobius (Sinuispineus) is morphologically similar to Lithobius (Monotarsobius) Verhoeff, 1905 but can be readily distinguished by the following characters: posterior spurs on the dorsal side of the femur of legs 15 in males are curved in *Sinuispineus* in contrast to straight in *Monotarsobius*; tarsal articulation ill-defined on legs 1–13 versus very faint or indistinct in *Monotarsobius*; 9–13 ocelli versus ocelli generally few, 1+1-1+11, in *Monotarsobius*.

#### Lithobius (Sinuispineus) sinuispineus sp. nov.

Taxon classificationAnimaliaLithobiomorphaLithobiidae

73D35598-8C18-5FA1-9C44-23AAB04385D5

http://zoobank.org/43E532BB-A9DC-43CF-A352-9DADB204C9BD

Figs 1 , 2A–L

##### Diagnosis.

Antennae 20–25 articles; ocelli usually nine on each side, in three irregular rows; posterior two ocelli comparatively large; Tömösváry’s organ larger than adjacent ocelli. Commonly 2+2 coxosternal teeth; porodonts lying posterolateral to lateral-most tooth. Coxal pore formula 3–5 in one row. Tarsal articulation ill-defined on legs 1–13, well-defined on legs 14 and 15. Female gonopods with 2+2 moderately small, coniform spurs; male gonopods short and small. Legs 15 are considerably modified in males: posterior spurs on dorsal side of femur of male legs 15 curving backward near base.

##### Material examined.

**Figure 1.**
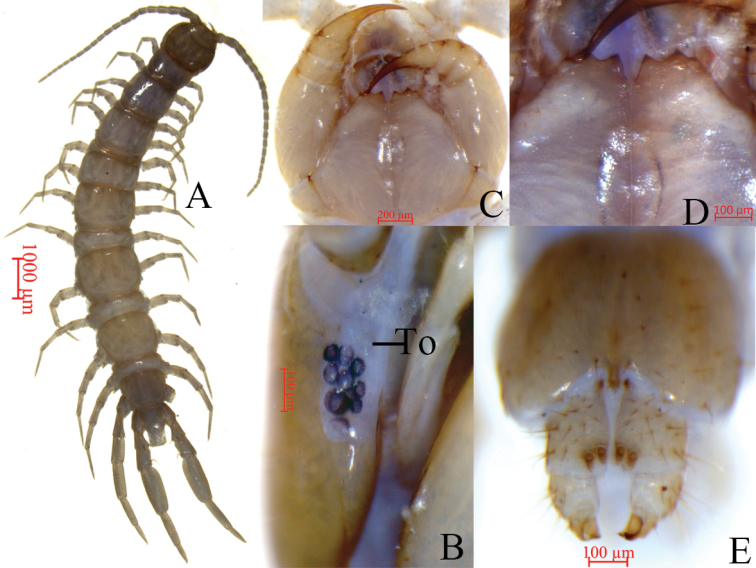
Lithobius (Sinuispineus) sinuispineus sp. nov. (holotype **A–D**, paratype female **E**) **A** habitus, dorsal view **B** ocelli and Tömösváry’s organ (To), lateral view **C** cephalic plate, ventral view **D** forcipular coxosternite, ventral view **E** posterior segments and gonopods of female, ventral view.

***Holotype***: ♂ (LS01-1) (Fig. 1A); body 11.56 mm long; cephalic plate 1.09 mm long, 1.24 mm wide. South East China, Huanggangshan, Wuyishan National Nature Reserve, Wuyishan County, Nanping City, Fujian Province, 27°52.025'S, 117°51.030'E, 544 m, 15 August 2010, leg. F. Zhang, H. Ma. ***Paratypes***: 1♀, 1♂ (LS01-1), same locality and date as holotype.

##### Other material examined.

2 ♂♂ (LS01-2), South East China, Yulinting, Wuyishan National Nature Reserve, Wuyishan County, Nanping City, Fujian Province, 27°40.917'S, 117°56.030'E, 462 m, 8 August 2010, leg. F. Zhang, H. Ma.

##### Description.

Body 11.6–15.2 mm long; cephalic plate 1.1–1.3 mm long, 1.2–1.4 mm wide.

***Colour***: antennae pale brown to pale greyish yellow from base to end; tergites pale brown, pleural region pale lavender, and sternites pale greyish yellow; basal parts of forcipules, forcipular coxosternite, and SS14 and 15 darker; coxa, trochanter, prefemur, femur, and tibia of all legs pale lavender; tarsus 1 pale brown; tarsus 2 pale yellow-brown in all legs.

***Antennae***: 25 articles in female, 20 articles in male (Fig. 1A). Length of first antennal article approximately equal to width of base; length of remaining articles longer than wide. Second article thicker and longer than other articles: from second article, each article gradually shortened, and distalmost articles still considerably longer than wide, 3.8–4.3 times as long as wide. Abundant setae on antennal surface, less so on basal articles; density of setae to approximately fourth article gradually increasing, then more or less constant.

***Cephalic plate*** smooth, convex, slightly wider than long; tiny setae emerging from pores scattered very sparsely over the whole surface; frontal marginal ridge with shallow anterior median furrow; short to long setae scattered along the marginal ridge of the cephalic plate; lateral marginal ridge discontinuous, posterior margin continuous, wider than lateral marginal ridge (Fig. 1A).

Nine, oval to rounded ocelli on each side, from small to large, arranged in three irregular rows; posterior two ocelli comparatively large. Ventral ocelli slightly smaller than the dorsal, domed, translucent, and usually darkly pigmented (Fig. 1B).

Tömösváry’s organ (Fig. 1B-To) near ocelli, at anterolateral margin of cephalic plate; moderately larger than adjoining ocelli; surrounding sclerotised area not obvious.

Coxosternite subtrapezoidal (Fig. 1C); anterior margin narrow; lateral margins slightly longer than medial margins. Median diastema moderately deep, narrowly V-shaped; anterior margin with 2+2 acute triangular teeth. Porodonts thicker, lying posteriolaterally to lateral-most tooth (Fig. 1C). Long, scattered setae on ventral side of coxosternite; longer setae near dental margin.

All tergites smooth, without wrinkles, tiny setae emerging from pores scattered sparsely over entire surface; near margin with few long setae. T1 narrower postero-laterally than antero-laterally, generally trapezoidal; T1 and T3 narrower than cephalic plate; T3 wider than T1; T8 widest. Lateral marginal ridges of all tergites continuous. Posterior margin of TT1 and 6 straight; TT 3, 5, 8, 10, 12, 14 slightly concave. Posterior angles of tergites rounded, without triangular projections. Miniscule setae scattered sparsely over the surface; one thick and long setae on both anterior angles of each tergite.

Posterior side of sternites narrower than anterior, generally trapezoidal, smooth. Setae emerging from sparsely scattered pores on the surface and lateral margin, very few long setae scattered sparsely among them. One comparatively long thick seta on both anterior angles of each sternite; more setae on surface of anterior and middle parts than posterior part of each sternite.

***Legs*** relative robust; tarsi fused on legs 1–13; well-defined on legs 14–15. All legs with fairly long, curved claws. Legs 1–12 with anterior and posterior accessory spurs; anterior accessory spurs moderately long and slender, forming a moderately small angle with claw; posterior accessory spurs slightly more robust, forming a comparatively large angle with claw; legs 13 with anterior accessory spurs; legs 14 and 15 lacking accessory spurs. Short to long setae sparsely scattered over surface of coxa, trochanter, prefemur, femur, and tibia of all legs; more setae on tarsal surface; setae on dorsal surface of tarsus slightly shorter than ventral surface. Legs 14 and 15 in female thicker than anterior pairs; legs 15 in male considerably thicker and stronger than anterior pairs. Leg plectrotaxy presented in Tables 1 and 2.

**Table 1.** Leg plectrotaxy of Lithobius (Sinuispineus) sinuispineus sp. nov. (female). Letters in brackets indicate variable spines.

Legs	Ventral					Dorsal				
	C	Tr	P	F	Ti	C	Tr	P	F	Ti
1			p	am	am			mp	ap	a
2			mp	am	am			mp	ap	(a)p
3–9			mp	amp	am			mp	ap	ap
10			mp	amp	am			(a)mp	ap	ap
11			mp	amp	am			amp	ap	ap
12			(a)mp	amp	am			amp	ap	ap
13		m	amp	amp	am		a	amp	ap	ap
14		m	amp	amp	am		a	amp	ap	p
15		m	amp	amp	a		a	amp	ap	

**Table 2.** Leg plectrotaxy of Lithobius (Sinuispineus) sinuispineus sp. nov. (male). Letters in brackets indicate variable spines.

Legs	Ventral					Dorsal				
	C	Tr	P	F	Ti	C	Tr	P	F	Ti
1			p	am	am			mp	ap	a
2			p	am (p)	am			mp	ap	ap
3–6			mp	amp	am			mp	ap	ap
7–11			mp	amp	am			amp	ap	ap
12			(a)mp	amp	am			amp	ap	ap
13		m	amp	amp	am		a	amp	ap	ap
14		m	amp	amp	am		a	amp	ap	p
15		m	amp	amp	a		a	amp	ap	p

Coxal pores 3–5 in a row, round or slightly oval, size of pores varies greatly from 19.3 μm to 48.7 μm; coxal pore field set in a relatively deep groove; coxal pore-field fringe with prominence; prominence with 4–8 moderately long setae sparsely scattered over the surface.

**Figure 2.**
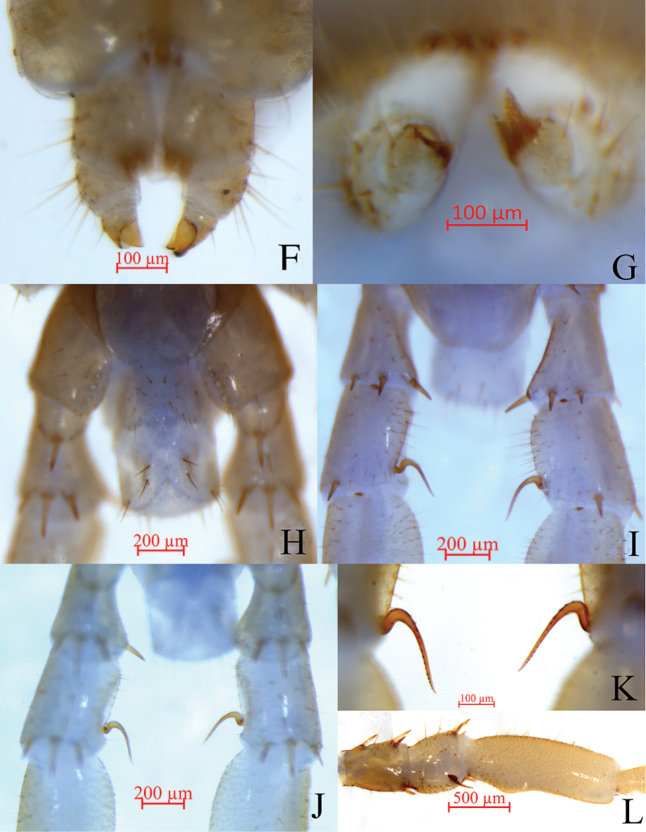
Lithobius (Sinuispineus) sinuispineus sp. nov. (paratype female **F, G**; holotype **H–L**) **F** claw of female gonopod, ventral view **G** claw of female gonopod, dorsal view **H** posterior segments and gonopods in male, ventral view **I** posterior spurs of the femur of legs 15, dorsal view **J** posterior spurs of the femur of legs 15, ventral view **K** posterior spurs of the femur of legs 15, ventral view, showing minor teeth **L** tibia of legs 15 raised inward.

**Female.** S15 anterior margin broader than posterior, generally trapezoidal, postero-medially slightly convex. Short to long setae sparsely scattered on S15 surface. Surface of lateral sternal margin of genital segment well chitinised, posterior margin of genital sternite deeply concave between condyles of gonopods, except for a small, median, tongue-shaped bulge. Relatively long setae scattered over ventral surface of genital segment, slightly more setae near S15. Gonopods: first article fairly broad, bearing 13 short to moderately long setae, arranged in three irregular rows; with 2+2 small coniform spurs; inner spur slightly smaller than the outer (Fig. 1E); second article ventrally with five or seven long setae, arranged in two irregular rows; third article ventrallywith two long setae, with a bidentate apical claw (Fig. 2F, G).

**Male.** S15 posterior margin narrower than anterior, postero-medially straight; sparsely covered with long setae, more than the anterior; sternite of genital segment slightly smaller than in female, usually weaker sclerotised; posterior margin deeply concave between gonopods, without medial bulge. Long setae scattered on ventral surface of genital segment; fewer setae near S15, fringed with 16–18 longer setae along posterior margin. Gonopods short, appearing as small finger-like bulges, with two long setae, apically slightly sclerotised (Fig. 2H). Legs 15 prominently modified, very thick; prefemur and femur very short, of unusual thickness (Fig. 2H–L); posterior spines of dorsal end of femur curved backward toward base of tibial segment at approximately 60° angle (Fig. 2H–K); anterior tibia raised inwards medially (Fig. 2L).

#### Lithobius (Sinuispineus) minuticornis sp. nov.

Taxon classificationAnimaliaLithobiomorphaLithobiidae

933C3EEA-E662-5AA2-ACAB-85A2E5EAE2E7

http://zoobank.org/BBC887BC-B357-4815-B5A2-B46B1431147A

Figs 3 , 4 A–K

##### Diagnosis.

Antennae composed of 20–23 articles; ocelli 8–10 on each side, arranged in three irregular rows; posterior two ocelli comparatively large. Tömösváry’s organ larger than adjacent ocelli. Commonly 2+2 coxosternal teeth; porodonts lying posterolateral to the lateral-most tooth. Coxal pore formula 3–6, usually 4443 or 5555. Female gonopods with 2+2 moderately small, coniform spurs; male gonopods short and small. Legs 15 considerably modified; prefemur and femur markedly strong, slightly raised inwards, and posterior spurs on dorsal side of femur of male legs 15 curved backward at base of tibia at no more than 45° angle.

##### Etymology.

The specific name refers to the small, backward-curved posterior spines on the dorsal end of the femur.

##### Material examined.

**Figure 3.**
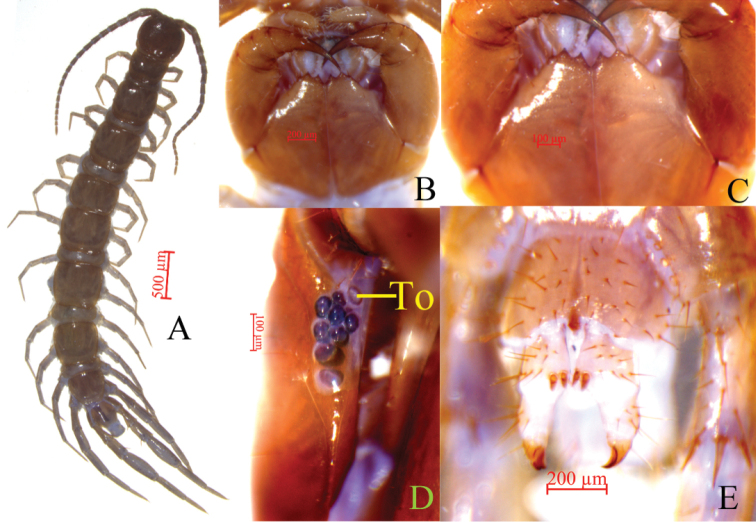
Lithobius (Sinuispineus) minuticornis sp. nov. (holotype **A–D**, paratype female **E**) **A** habitus, dorsal view **B** cephalic plate, ventral view **C** forcipular coxosternite, ventral view **D** ocelli and Tömösváry’s organ (To), lateral view **E** posterior segments and gonopods of female, ventral view.

***Holotype***, ♂ (LS02-1) (Fig. 3A), 12.31 mm long, cephalic plate 1.31 mm long, 1.47 mm wide, Central China, Zhenlei Mountain Forest Park, Pingqiao County, Xinyang City, Henan Province, 32°04.445'S, 114°08.403'E, 256 m a.s.l. August 27, 2017, S. Pei, H. Ma leg. ***Paratypes***, 65 ♀♀, 50 ♂♂ (LS02-1), same date and locality as holotype.

##### Description.

***Body*** 10.8–16.7 mm long; ***cephalic plate*** 1.0–1.5 mm long, 1.1–1.5 mm wide.

***Colour***: antennae brown to pale yellow from basal to proximal; distal-most article yellow-brown; tergites brown; pleural region and sternites pale greyish yellow; basal and proximal parts of forcipules, forcipular coxosternite, and SS14 and 15 darker; coxa, trochanter, prefemur, femur, and tibia of all legs bluish; tarsus 1 yellow; tarsus 2 yellow-brown.

***Antennae***: 20–23 articles, commonly 20 articles (Fig. 3A). Except that length of first antennal article is approximately equal to width of base, length of remaining articles larger than width. Moreover, second article thicker and longer than other articles. Beginning with second article, each article gradually shortened; distal-most article considerably longer than width, 3.0–3.9 times longer as wide. Setae abundant on antennal surface; less so on basal articles; density of setae gradually increase to approximately fifth article, then more or less constant.

***Cephalic plate*** smooth, convex, slightly longer than wide; tiny setae emerging from pores scattered very sparsely over the whole surface; frontal marginal ridge with shallow anterior median furrow; short to long setae scattered along marginal ridge of cephalic plate; lateral marginal ridge discontinuous, posterior margin continuous, wider than lateral marginal ridge; middle of the posterior edge straight.

Eight to ten (commonly nine) oval to rounded ocelli on each side, from small to large, in three irregular rows; posterior two ocelli comparatively large. Ventral ocelli slightly larger than dorsal ocelli, domed, translucent, and usually darkly pigmented (Fig. 3D).

Tömösváry’s organ (Fig. 3D-To) near the ocelli, at anterolateral margin of cephalic plate moderately larger than the adjoining ocelli, surrounding sclerotised area not obvious.

Coxosternite subtrapezoidal (Fig. 3B, C); anterior margin narrow; lateral margins slightly longer than medial margins; median diastema moderately deep, narrowly V-shaped; anterior margin with 2+2 slightly larger, acutely triangular teeth; porodonts thicker, with prominent basal protuberance, lying posterior-lateral to lateral-most tooth (Fig. 3B, C); long, scattered setae on ventral side of coxosternites; longer setae near dental margin.

All tergites smooth, without wrinkles; dorsum slightly convex; tiny setae emerging from sparsely scattered pores over entire surface; near margin bearing a few long setae; TT1 and 14 narrower postero-laterally than antero-laterally, generally trapezoidal; T1 narrower than cephalic plate; T3 approximately equal to cephalic plate; T10 widest. Lateral marginal ridges of all tergites continuous. Posterior margins of TT1, 3, 5, and 7 straight; TT8, 10, 12, and 14 slightly concave. Posterior angles of tergites rounded, without triangular projections. Miniscule setae sparsely scattered over surface.

Posterior side of sternites narrower than anterior, generally trapezoidal, smooth; SS6, 7, 8, 9, and 10 more wider, setae emerging from sparsely scattered pores on surface and lateral margin, with very few long setae sparsely scattered among of them; 2–4 comparatively thick, long setae on both of anterior angles of each sternite; one or two comparatively long, thick setae on both posterior angles of each sternite; more setae on surface of anterior and middle parts than posterior part of each sternite.

***Legs*** relative robust; tarsi fused on legs 1–13; tarsi well-defined on legs 14–15; all legs with fairly long curved claws; legs 1–13 with anterior and posterior accessory spurs; anterior accessory spurs moderately long and slender, forming a moderately small angle with claw; posterior accessory spurs slightly more robust, forming a comparatively large angle with claw; legs 14 and 15 lacking accessory spurs. Short to long setae sparsely scattered over surface of coxa, trochanter, prefemur, femur, and tibia of all legs; more setae on tarsal surface; setae on dorsal tarsal surface slightly shorter than on ventral surface. Legs 14 and 15 in female thicker than anterior pairs; legs 15 in male considerably thicker and stronger than anterior pairs. In females, tarsus2 62.9%–78.8% length of tarsus 1 on legs 15; tarsus 2 5.1–6.5 times longer than its maximum width. In males, tarsus 2 61.3%–70.1% length of tarsus 1 on legs 15; tarsus 2 is 3.5–4.7 times longer than its maximum width. Leg plectrotaxy as in Tables 3 and 4.

Coxal pores 3–6, in a row, round or slightly oval, greatly variable in size from 18.6 μm to 50.7 μm; coxal pore field set in a relatively deep groove; coxal pore-field fringe with prominence; prominence with 8–12 moderately long setae sparsely scattered over surface.

**Table 3.** Leg plectrotaxy of Lithobius (Sinuispineus) minuticornis sp. nov. (female). Letters in brackets indicate variable spines.

Legs	Ventral					Dorsal				
	C	Tr	P	F	Ti	C	Tr	P	F	Ti
1			p	amp	am			mp	a(p)	a
2			p	amp	am			mp	ap	a
3			p	amp	am			mp	ap	ap
4–10			mp	amp	am			mp	ap	ap
11			(a)mp	amp	am			amp	ap	ap
12			amp	amp	am			amp	p	ap
13		m	amp	amp	am			amp	p	ap
14		m	amp	amp	(m)	a		amp	p	p
15		m	amp	am		a		amp	ap	

**Table 4.** Leg plectrotaxy of Lithobius (Sinuispineus) minuticornis sp. nov. (male). Letters in brackets indicate variable spines.

Legs	Ventral					Dorsal				
	C	Tr	P	F	Ti	C	Tr	P	F	Ti
1			(p)	am	am			mp	(a)p	a
2			mp	am	am			mp	ap	a
3			mp	am	am			mp	ap	ap
4–7			mp	am	am			mp	ap	ap
8–10			m	amp	am			mp	ap	ap
11			m	amp	am			amp	ap	ap
12			(a)mp	amp	am			amp	(a)p	ap
13		m	amp	amp	am			amp	p	ap
14		m	amp	amp	m	a		amp	p	
15		m	amp	am		a		amp	(a)p	

**Figure 4.**
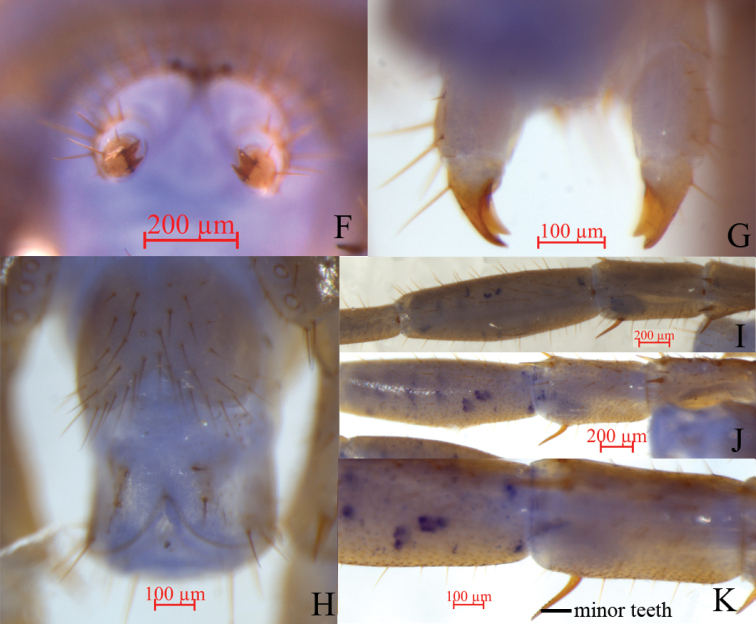
Lithobius (Sinuispineus) minuticornis sp. nov. (paratype female **F, G**; holotype **H–K**) **F** bidentate apical claw of female gonopod, dorsral view **G** bidentate apical claw of female gonopod, ventral view **H** posterior segments and gonopods in male, ventral view **I** femur and tarsus of legs 15, dorsral view **J** femur and tarsus of legs XV15, ventral view **K** posterior spurs of femur of legs 15, ventral view, showing minor teeth.

**Female.** S15 anterior margin broader than posterior, generally trapezoidal, both of anterior and posterior angles generally rounded, posteriomedially straight, short to long setae sparsely scattered on S15 surface. Sternite of genital segment longer than wide, with surface of its lateral sternal margin well chitinised and posterior margin deeply concave between condyles of gonopods, except for a small, median, approximately rhombic bulge. Relatively long setae scattered over ventral surface of genital segment and slightly more setae near S15. Gonopods: first article fairly broad, bearing 22–24 moderately long setae arranged in three irregular rows; with 2+2 small coniform spurs, inner spur slightly smaller than outer spurs (Fig. 3E); second article ventrally with 5–7 long setae, arranged in three irregular rows; third article ventrally with one long and one short setae and with a bidentate apical claw (Fig. 4F, G).

**Male.** S15 posterior margin narrower than anterior, with both posterior angles rounded; posterior-medially straight, sparsely covered with 31–33 long setae, more than on anterior; sternite of genital segment slightly smaller than in female, usually less sclerotised; posterior margin deeply concave between gonopods, without medial bulge. Long setae scattered on ventral surface of genital segment; fewer setae near S15; fringed with 8–16 longer setae along posterior margin. Gonopods short, appearing as small finger-like bulges, without setae, apically slightly sclerotised (Fig. 4H).

***Legs*** 15 prominently developed, very thick; prefemur and femur very short (Fig. 4I–K), posterior spines of dorsal base of femur curved backwards toward base of tibial segment at no more than 45° angle; anterior tibia raised medially inwards. Distal-most anterior spines of dorsal of coxa bearing one or two small teeth; distal-most middle spines of ventral trochanter with a small tooth; posterior part of prefemur considerably larger than anterior part; distal-most part of anterior, middle, and posterior spines of ventral prefemur with 2–4 small teeth; distal-most anterior, middle, and posterior spines of dorsal prefemur with 1–3 small teeth, with both left and right posterior spines arranged opposite each other; femur markedly thick, slightly raised inwards, with posterior spines of dorsal femur curving backwards towards base of tibial segment at no more than a 45° angle (Fig. 4I–K); distal-most anterior and middle spines of ventral femur with two or three small teeth.

##### Habitat.

The specimens were collected in a *Larix* forest at approximately 200 m above sea level. Specimens were living in moderately moist places under roadside stones and litter on the forest floor.

## Discussion

The two new species resemble each other in having the antennae with commonly 20 articles, 8–10 ocelli on each side arranged in three irregular rows, the posterior two ocelli comparatively large, the Tömösváry’s organ larger than the adjoining ocelli, 2+2 prosternal teeth, a coxal pore formula of 3–5, and female gonopods with 2+2 coniform spurs. However, they can be distinguished easily by the following characters. T10 is the widest tergite in *S. minuticornis* instead of T8 in *S. sinuispineus*. The posterior spines of the dorsal base of femur curve backwards towards the base of tibial segment at an angle of no more than 45° in *S. minuticornis* in contrast to more than 45° in *S. sinuispineus*. The dorsal plectrotaxy of legs 13 in *S. minuticornis* is 0-0-3-1-2, compared to 1-0-3-2-2 in *S. sinuispineus*. Anterior spines are lacking on both the dorsal and the ventral sides of the tibia of legs 14 and 15 in *S. sinuispineus* vs. present in *S. minuticornis*. Legs 13 having posterior accessory spurs in *S. minuticornis* rather than lacking in *S. sinuispineus*.

## Supplementary Material

XML Treatment for Sinuispineus

XML Treatment for Lithobius (Sinuispineus) sinuispineus

XML Treatment for Lithobius (Sinuispineus) minuticornis
